# Elucidating Binding Sites and Affinities of ERα Agonists and Antagonists to Human Alpha-Fetoprotein by In Silico Modeling and Point Mutagenesis

**DOI:** 10.3390/ijms21030893

**Published:** 2020-01-30

**Authors:** Nurbubu T. Moldogazieva, Daria S. Ostroverkhova, Nikolai N. Kuzmich, Vladimir V. Kadochnikov, Alexander A. Terentiev, Yuri B. Porozov

**Affiliations:** 1 Laboratory of Bioinformatics, I.M. Sechenov First Moscow State Medical University (Sechenov University), 119991 Moscow, Russia; daria.ostroverkhova@gmail.com (D.S.O.); nkuzmich@gmail.com (N.N.K.); yuri.porozov@gmail.com (Y.B.P.); 2 Department of Bioengineering, M.V. Lomonosov Moscow State University, 119991 Moscow, Russia; 3 Department of Drug Safety, I.M. Smorodintsev Research Institute of Influenza, WHO National Influenza Centre of Russia, 197376 Saint Petersburg, Russia; 4 Department of Food Biotechnology and Engineering, Saint Petersburg National Research University of Information Technologies, Mechanics and Optics, 197101 Saint-Petersburg, Russia; vlkadochnikov@gmail.com; 5 Deparment of Biochemistry and Molecular Biology, N.I. Pirogov Russian National Research Medical University, 117997 Moscow, Russia; aaterent@inbox.ru

**Keywords:** alpha-fetoprotein, estrogens, selective estrogen receptor modulators, homology-based modeling, molecular docking, protein–ligand interaction, amino acid substitutions

## Abstract

Alpha-fetoprotein (AFP) is a major embryo- and tumor-associated protein capable of binding and transporting a variety of hydrophobic ligands, including estrogens. AFP has been shown to inhibit estrogen receptor (ER)-positive tumor growth, which can be attributed to its estrogen-binding ability. Despite AFP having long been investigated, its three-dimensional (3D) structure has not been experimentally resolved and molecular mechanisms underlying AFP–ligand interaction remains obscure. In our study, we constructed a homology-based 3D model of human AFP (HAFP) with the purpose of molecular docking of ERα ligands, three agonists (17β-estradiol, estrone and diethylstilbestrol), and three antagonists (tamoxifen, afimoxifene and endoxifen) into the obtained structure. Based on the ligand-docked scoring functions, we identified three putative estrogen- and antiestrogen-binding sites with different ligand binding affinities. Two high-affinity binding sites were located (i) in a tunnel formed within HAFP subdomains IB and IIA and (ii) on the opposite side of the molecule in a groove originating from a cavity formed between domains I and III, while (iii) the third low-affinity binding site was found at the bottom of the cavity. Here, 100 ns molecular dynamics (MD) simulation allowed us to study their geometries and showed that HAFP–estrogen interactions were caused by van der Waals forces, while both hydrophobic and electrostatic interactions were almost equally involved in HAFP–antiestrogen binding. Molecular mechanics/Generalized Born surface area (MM/GBSA) rescoring method exploited for estimation of binding free energies (ΔG_bind_) showed that antiestrogens have higher affinities to HAFP as compared to estrogens. We performed in silico point substitutions of amino acid residues to confirm their roles in HAFP–ligand interactions and showed that Thr132, Leu138, His170, Phe172, Ser217, Gln221, His266, His316, Lys453, and Asp478 residues, along with two disulfide bonds (Cys224–Cys270 and Cys269–Cys277), have key roles in both HAFP–estrogen and HAFP–antiestrogen binding. Data obtained in our study contribute to understanding mechanisms underlying protein–ligand interactions and anticancer therapy strategies based on ERα-binding ligands.

## 1. Introduction

Alpha-fetoprotein (AFP) is a major mammalian embryo-specific and tumor-associated protein recognized as a “golden standard” among cancer biomarkers used in clinical practice [1]. The *AFP* gene is expressed during embryonic development by the liver and yolk sac and is down-regulated soon after the birth to be re-expressed in adult patients with hepatocellular carcinoma (HCC) [2,3,4]. Biological roles of AFP during embryonic development and tumor growth have long been investigated; however, they are still not fully understood [5]. Experimental data evidence capability of both native and recombinant AFP to regulate cell proliferation and immune response [6,7,8], as well as to bind and transport a variety of hydrophobic ligands, such as estrogens, fatty acids, and drugs, suggesting that these functions have a role during embryo- and carcinogenesis [9,10,11]. 

The estrogen-binding capability of AFP can contribute to a decrease in concentration of free active forms of estrogens below the levels necessary to activate the estrogen receptors (ERs) ERα and ERβ in target cells. Indeed, AFP has been reported to inhibit estrogen receptor (ER)-positive human MCF-7 and MTW9A rat mammary cancer growth, which is associated with the interaction between AFP and 17β-estradiol [12,13]. Additionally, synthetic human AFP (HAFP)-derived peptides cause inhibition of uterine estrogen-dependent cell proliferation and a simultaneous increase in the antitumor effect of tamoxifen [14]. Furthermore, high HAFP concentrations in the maternal blood serum during second and third trimesters of pregnancy correlate with reduced ER-positive breast cancer risk [15].

The canonical estrogen-signaling pathway starts from ligand binding to ERs, which causes conformational changes, dimerization, and activation of the receptor [16]. Further, the activated receptor–hormone complex is translocated into the nucleus, where it binds to specific sequences of the estrogen responsive element (ERE) in the promoter region of target genes [17,18]. ERs recruit a variety of co-regulatory proteins, co-activators, and co-repressors, which can cause alterations in hormone–receptor complex formation and DNA-binding abilities [19]. 

Natural estrogen, 17β-estradiol (E2), is the most potent female sex steroid hormone as compared to its metabolites, estrone (E1) and estriol (E3), in regulating cell proliferation, differentiation, and homeostasis in reproductive, skeletal, cardiovascular, and neural tissues [20,21,22]. Estimation of binding affinities of 125 ER ligands, both natural and chemically synthesized, performed using competitive ligand binding assay, demonstrated that certain structural features of estrogens such as the presence of aromatic ring may be important for their binding efficacy to ERs [23]. However, various endocrine disruptors can exert structural similarity to steroid hormones and can directly compete with estrogens for their ER-binding sites.

While estrogens are considered to be ER agonists, numerous selective estrogen receptor modulators (SERMs) can serve as both ER agonists and antagonists in a tissue- or promoter-specific and dose-dependent manner [24,25]. Among SERMs, tamoxifen is the most commonly used non-steroidal antitumor drug for breast cancer therapy. Tamoxifen and its derivatives afimoxifene (4-hydroxytamoxifen, 4OHT) and endoxifen (4-hydroxy-*N*-desmethyl-tamoxifen) are type 1 antiestrogens, which can act as both agonists and antagonists [26]. Structurally, tamoxifen is similar to diethylstilbestrol (DES), a synthetic non-steroidal estrogen and pure agonist recognized as a teratogen and carcinogen. However, unlike DES, tamoxifen has an additional aromatic ring and a side chain (trans-isomer) with *N*-linked methyl groups [27]. Afimoxifene and endoxifen are key active metabolites of tamoxifen and experimentally have been shown to possess higher affinity and specificity to ERα as compared to tamoxifen [28].

The ability of hormone-binding transport proteins to interfere with ER–ligand interactions dictates the importance of investigation of binding affinities of both estrogens and ER disruptors to both ERs and estrogen-binding transport proteins, such as AFP. Rodent AFPs have been experimentally evidenced to bind free estrogens, while HAFP has been shown to bind only immobilized estrogens [29,30]. Using chimeric human-rat AFP, the existence of two types of estrogen-binding sites (high-affinity and low-affinity) in rat AFP has been proposed [31,32]. However, experimentally obtained three-dimensional (3D) structures of AFP do not exist for any biological species in the Protein Data Bank (PDB) database [33], either with or without ligands. This dictates the necessity to create a 3D model of AFP followed by molecular docking of ligands into the obtained structure to investigate AFP–ligand binding affinities and mechanisms underlying protein–ligand interactions at an atomic level. This might contribute to understanding the role of estrogen and antiestrogen binding to transport proteins in endocrine system function, in both normal and pathological states.

HAFP contains 609 amino acid residues and is composed of three homologous domains, namely domains I (aa 19-210), II (aa 211-402), and III (aa 402-601). It belongs to the serum albumin (SA) family, which includes two more proteins, namely alpha-albumin (afamin) and vitamin D-binding protein (VTDB). Members of this family are localized in tandem arrangement in the q11-q13 region of chromosome 4 and share a high degree of sequence similarity [34]. The sufficient degree of sequence identity (up to 40%) between AFP and SA makes it possible to construct a 3D model of AFP based on the homology with SA, for which experimentally obtained crystal structures can be found in the PDB database [33]. Previously, we used this approach to build a 3D structure of HAFP using homology-based modeling and investigated binding modes of DES, a validated estrogen disruptor [35].

Later, Shen and co-workers built a homology-based 3D model of rat AFP to elucidate binding modes and to estimate binding affinities of a variety of estrogens and estrogen disruptors [36]. The ability of rat AFP to undergo conformational changes during ligand binding and to accommodate numerous structurally and physicochemically diverse compounds has been observed. Additionally, testing of a large set of structurally different chemical compounds from 13 structural categories was performed, revealing 53 rat AFP binders and 72 non-binders [37]. 

In the present study, we performed homology-based modeling of a 3D structure of HAFP with the aim of performing molecular docking of three agonists and three antagonists of ERα to the obtained structure to elucidate binding modes and the topology of estrogen- and antiestrogen-binding sites. The molecular mechanics/Generalized Born surface area (MM/GBSA) rescoring method based on molecular dynamics (MD) simulation was exploited to assess affinities of estrogens to HAFP compared to antiestrogens. Further, we performed in silico point substitutions of amino acid residues that are presumably involved in estrogen binding to confirm their roles in protein–ligand interactions. We found that there are three principal estrogen- and antiestrogen-binding sites in HAFP. Two of them are high-affinity sites located (i) in the tunnel formed within HAFP subdomains IB and IIA, and (ii) on the opposite side of the molecule in a groove originating from the cavity formed by the U-shaped AFP structure, which was in accordance with experimental data [38]. The third site is a low-affinity binding site located at the bottom of the cavity. Investigation of the binding site geometries allowed us to elucidate amino acid residues involved in HAFP–ligand binding and forces involved in HAFP–estrogen and HAFP–antiestrogen interactions. We showed that antiestrogens have higher affinity to HAFP as compared both natural and synthetic estrogens, which may be dictated by differences in their structures and amounts of rotameric states.

## 2. Results

### 2.1. The Overall Architecture and Quality of Constructed HAFP 3D Model

**Figure 1:**
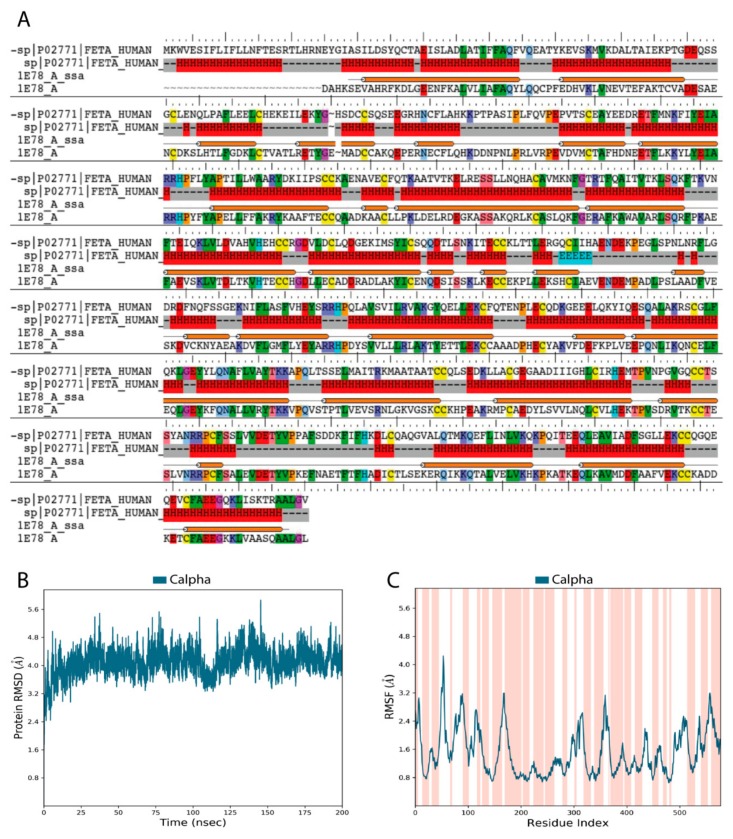
Validation of human alpha-fetoprotein (HAFP) three-dimensional (3D) model structure obtained based on homology with human serum albumin (HSA). (**A**) Target template sequence alignment. Secondary structure elements are indicated, represented predominantly by α-helices (H) and solvent accessible surface area (SASA). Identical residues are similarly colored according to their physicochemical properties. (**B**) RMSD plot obtained for Cα-atoms during 200 ns MD simulation shows the stability of the HAFP 3D structure. (**C**) RMSF plot (dark blue) superimposed on secondary structure elements (pink colored regions). Peaks correspond to flexible regions with irregular secondary structures.

Validation of our constructed 3D model of the HAFP structure showed that it was of high quality and reliability. Figure 1A depicts pairwise alignment between sequences of target proteins, HAFP retrieved from Uniprot knowledge base (ID: P02771), and human serum albumin (HSA), which was identified as a template for modeling and retrieved from the PDB database (ID: 1E78). The identity degree is equal to 39%, and secondary structure elements (SSE) are represented by α-helices and random coils with no β-strands, in accordance with experimental data [38]. Ramachandran maps generated by PROCHECK program verified proper stereo-chemical and conformational properties of amino acid residues in the modeled HAFP structure (Figure S1A). Indeed, 92.7% of all residues were located in favored regions, 6.8% of all residues were in additional allowed regions, while two residues, Ser84 and Glu565, were in generously allowed regions. Only one residue, Ala39, occurred in a disallowed region. Furthermore, a structural alignment score for the obtained model against the used template, 1E78, was very low at 0.002, along with a RMSD value of 0.196 Å.

Analysis of stereo-chemical parameters of the obtained structure (Figure S1B) showed that all residues have correct Cα-chirality, while only 1.2% of residues have incorrect absolute deviation from the mean *χ*1 value and 5% of residues have incorrect absolute deviation from the mean ω torsion. Estimation of water accessibility areas showed that α-helical regions are mostly buried, indicating their enrichment in hydrophobic residues. Additionally, G-factor values for the overall structure and the main chain bonds and angles, along with combinations of φ-ψ, χ1-χ2, and χ3-χ4, indicate the high quality of the model obtained in this study and its reliability for further usage.

Relaxation and optimization of the constructed HAFP model before ligand docking was performed using 200 ns MD simulation, which allowed further improvement of its quality. Indeed, RMSD values calculated for Cα-atoms during 200 ns MD simulation of the HAFP model were stabilized around 3.8–4.0 Å (Figure 1B). RMSF plots obtained for residues in the HAFP sequence allowed us to judge local fluctuations, which were not high and corresponded to regions with irregular structures and loops (Figure 1C). Importantly, a single peak with a RMSF value higher than 4.0 Å is located in domain IA of HAFP, which does not contain ligand binding residues. Additionally, the high quality of the HAFP 3D model was confirmed by analysis of the secondary structure element (SSE) composition for each 200 ns MD simulation trajectory frame, which showed its stability (Figure S1C). Superimposition of HAFP models before and after 200 ns MD simulation measured by RMSD alignment showed a sufficiently low alignment score of 0.457 and a RMSD of 3.371 Å. The optimized structure became more relaxed and expanded after optimization, as observed by the structure visualization using Maestro suite in the Schrödinger software.

**Figure 2:**
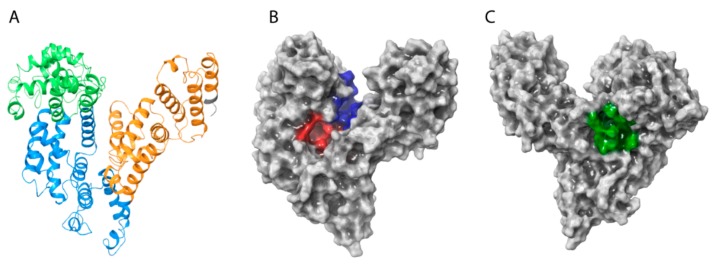
The overall architecture and domain organization of the HAFP 3D model structure. (**A**) Three-domain organization, where domains I, II, and III are shown in green, blue, and brown, respectively. (**B**) Topology of the entire HAFP surface in the vicinity of a tunnel (red) formed within subdomains IB and IIA and the inter-domain cavity (blue) formed between domains I and III. (**C**) A groove (green) originating from the cavity.

Analysis of the HAFP 3D model architecture showed its U-shaped structure, in which three domains and secondary structure elements represented by α-helices with no β-strands can be identified (Figure 2A). Additionally, visualization of the model surface by Maestro suite revealed an inter-domain cavity formed by HAFP domains I and III and a tunnel (Figure 2B) located between its subdomains IB and IIA. This tunnel has a length of about 29.3 Å and a diameter of about 9.25 Å in its narrowest part and of 15.24 Å in its widest segment. Furthermore, a groove originated from the cavity (Figure 2C) formed between domains I and III may be observed on the back side of the HAFP surface.

### 2.2. HAFP–Ligand-Docked Poses

**Figure 3:**
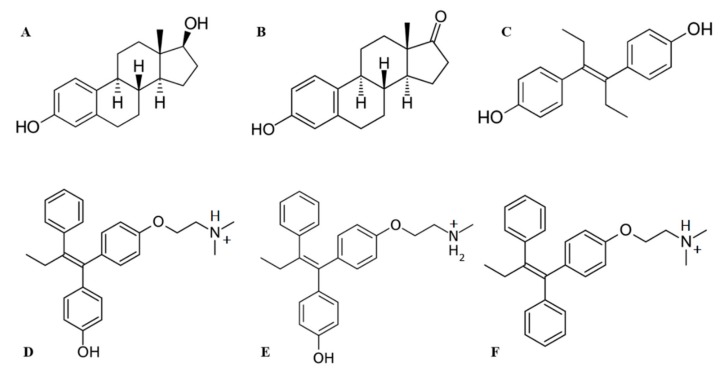
Structures of ligands used in this study: (**A**) 17β-estradiol, (**B**) estrone, (**C**) diethylstylbestrol, (**D**) afimoxifene, (**E**) endoxifen, (**F**) tamoxifen.

The molecular docking procedure was performed to search for putative binding sites of 6 ligands—3 estrogens and 3 antiestrogens (Figure 3)—in HAFP and to estimate the ligand binding modes. Ligand molecules extracted from Pubchem database were optimized and docked into the optimized HAFP modeled structure using a semi-flexible docking protocol, in which all possible rotations and all probable conformations of the ligand molecules were enabled. The SiteMap algorithm in Schrodinger software version 2018-2 allowed prediction of 14 potential binding sites in the HAFP 3D model structure that were suitable for occupancy by ligands due to non-covalent forces, such as hydrophobic and H-bond interactions. Further, 36 Å grids were generated and centered on a potential binding site, and all ligands were placed into each grid. In total, 84 ligand-docked complexes were obtained. 

The SiteMap algorithm is integrated with the Glide algorithm in Shrodinger software, and this enables not only identification but also evaluation of potential binding sites, along with their ranking based on calculation of ligand-docked scoring functions. Based on Glide gscore and emodel scoring functions, we elucidated sites that best accommodate ligands and selected ligands with the best binding affinities (Table 1). Gscore enables ranking of all ligands by their binding affinities to a definite site, while emodel allows comparison of sites by their ability to accommodate specific ligand. 

As shown in Table 1, ligand-docked functions show that for all ligands, both estrogens and antiestrogens have higher affinity to sites A and C as compared to site B. To confirm this and to identify dynamic HAFP–ligand binding modes and ligand binding affinities, we further carried out a 100 ns MD simulation study of the 12 best protein–ligand complexes, which were selected on the basis of the ligand-docked scoring functions. 

**Figure 4:**
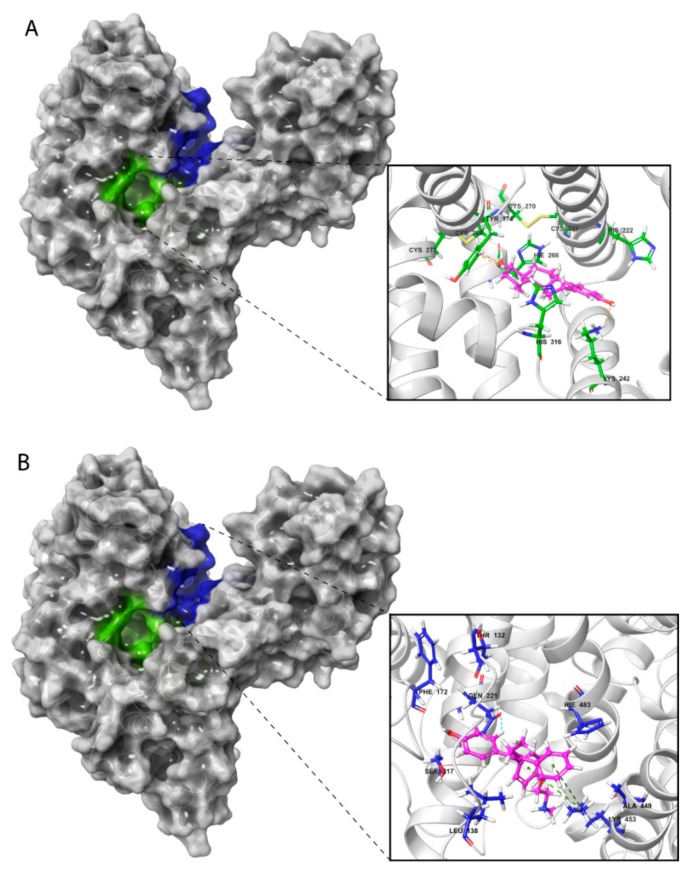

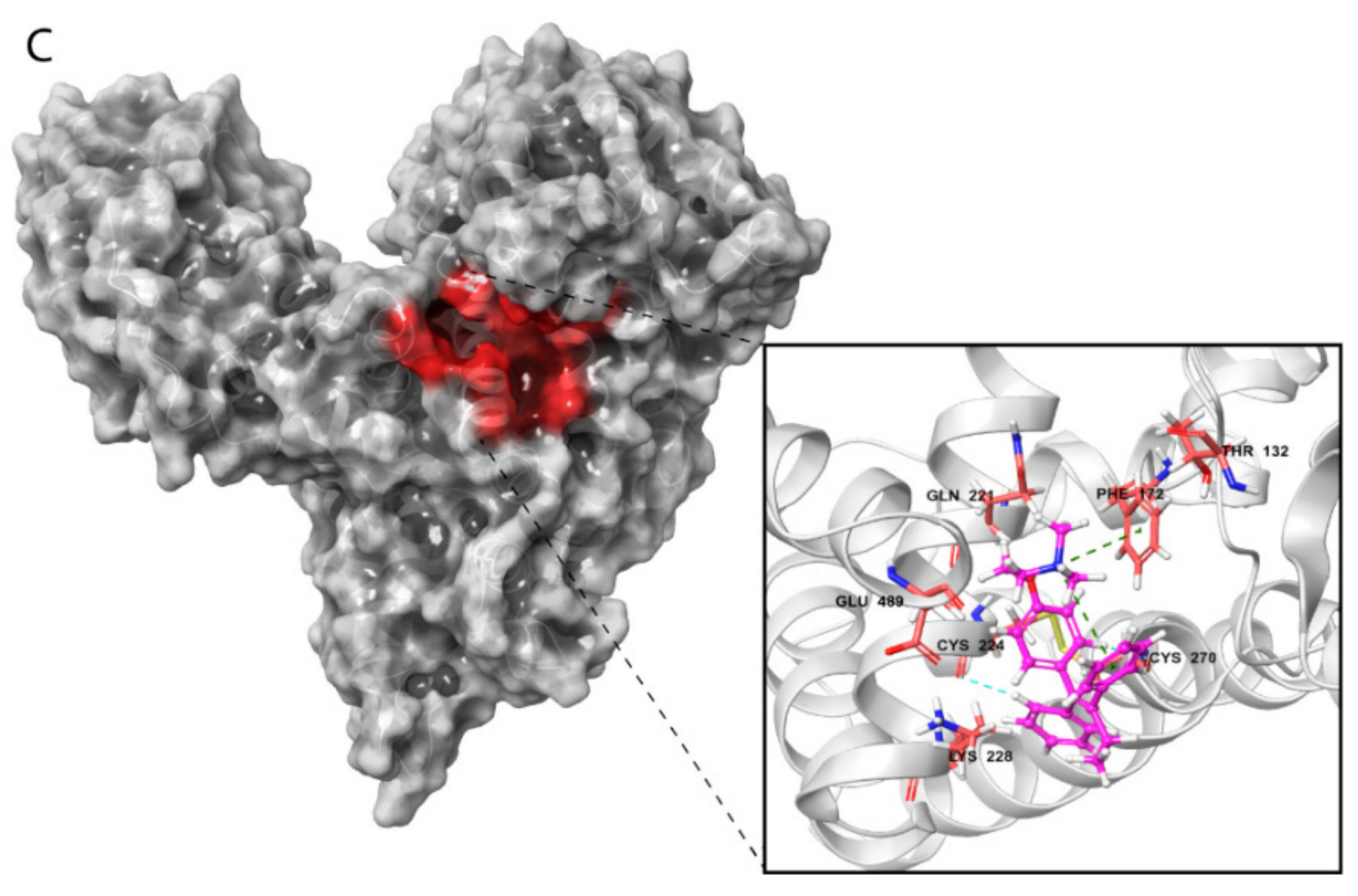
Amino acid residues involved in HAFP–ligand binding based on ligand-docked poses. (**A**) The 17β-estradiol in site A formed by the amino acid residues Tyr174, His222, Lys242, His266, and His316, along with the disulfides Cys224–Cys270 and Cys269–Cys277. (**B**) Afimoxifene in site B composed of Thr132, Leu138, Phe172, Ser217, Gln221, His483, Ala449, and Lys453. (**C**) Tamoxifen in site C, which contains Lys228 and Glu489 and can involve some residues from sites A and B.

Visualization of these complexes allowed identification of the three most potent estrogen-binding sites (designated as A and B) and antiestrogen-binding sites (designated as C) (Figure 4A–C). Site A was located in a tunnel formed between subdomains IB and IIA, site B was on the bottom of the cavity in a U-shaped HAFP structure, and site C was in a groove that originated from the cavity and that was located on the back side of the HAFP molecule.

### 2.3. Binding of Ligands to HAFP Studied by MD Simulation

#### 2.3.1. Stability of HAFP–Ligand Complexes

**Figure 5:**
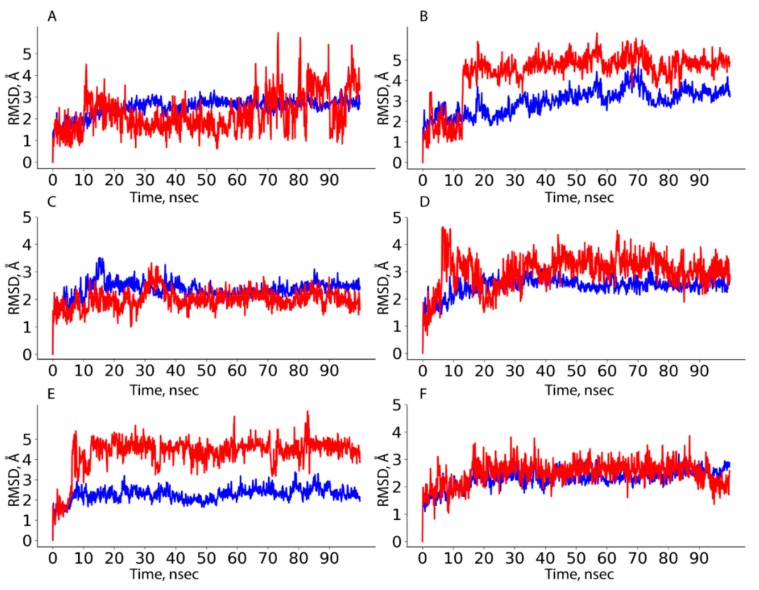
HAFP–ligand RMSD plots drawn during 100 ns MD simulation in sites with the most stable protein–ligand complexes: (**A**) HAFP–E2 complex in site B, (**B**) HAFP–E1 complex in site B, (**C**) HAFP–DES complex in site A, (**D**) HAFP–endoxifen complex in site A, (**E**) HAFP–afimoxifene complex in site C, (**F**) HAFP–tamoxifen complex in site C.

Estimation of the stability of HAFP–ligand complexes was done based on protein–ligand RMSD calculations. Figure 5 depicts representative RMSD plots for HAFP–ligand complexes optimized by 100 ns MD simulation. As shown in Figure 5A, protein conformation in the HAFP–E2 complex formed in site B remained stable during the 100 ns MD simulation, stabilizing at around 2.4–2.8 Å after the 20 ns simulation. Ligand RMSD values remain stable at about 2.0 Å, remaining lower than protein RMSD values until the 65 ns MD simulation, indicating that E2 remained in the binding site. As shown in Figure 5B, in the HAFP–E1 complex, ligand RMSD values sharply increased after 15 ns MD simulation indicating, that the ligand moves away from the binding site. As for the HAFP–DES complex, the protein–ligand RMSD plots show that the most stable interactions are observed in site A (Figure 5C). The affinity of endoxifen to HAFP was high in all three sites and was the best in site A (Table 2), in which protein–ligand RMSD remained stable during 100 ns MD simulations (Figure 5D). The best HAFP–afimoxifene and HAFP–tamoxifen complexes were in site C, showing sufficient stability during the 100 ns MD simulation (Figure 5E,F).

#### 2.3.2. Ligand Binding Affinities

To assess ligand binding affinities, the MM/GBSA rescoring method was implemented based on 100 ns MD simulation. For this purpose, we calculated MM/GBSA values for each of the protein–ligand RMSD snapshots obtained during the 100 ns MD simulation and then calculated average MM/GBSA values for each trajectory. Table 2 shows that MM/GBSA ΔG_bind_ values mainly decreased and consequently binding affinities increased after optimization of HAFP–ligand complexes. Exceptions are the HAFP–afimoxifene complex in site A and HAFP–endoxifen and HAFP–DES complexes in site C, in which MM/GBSA values did not decrease after optimization. This may be explained by both ligand motions during MD simulations and the local arrangements of amino acid residues in ligand binding sites. This can depend on both the structural features of a ligand (the presence of an extra OH group in afimoxifene, endoxifene, and DES) and the geometry of a binding site, such as a narrow tunnel (site A) or groove (site C), which create restrictions for conformational motions. To verify this, we further estimated dynamic changes in protein–ligand contacts and interaction modes followed by in silico substitutions of key amino acid residues presumably involved in protein–ligand interactions.

#### 2.3.3. HAFP–Ligand Interaction Modes

**Figure 6:**
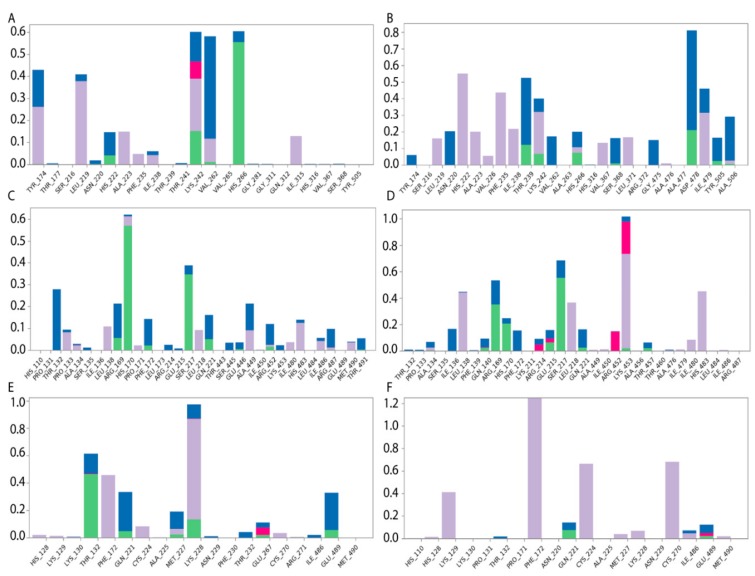
Diagrams of HAFP–ligand contacts in the most stable complexes with indications of amino acid residues and forces involved throughout 100 MD ns simulations. Protein–ligand interactions were categorized into four types: H-bonds (green), ionic (red), hydrophobic (purple), and water bridges (blue). (**A**) HAFP–E2 contacts in site A, (**B**) HAFP–DES contacts in site A, (**C**) HAFP–E1 contacts in site B, (**D**) HAFP–endoxifen contacts in site B, (**E**) HAFP–afimoxifene contacts in site C, and (**F**) HAFP–tamoxifen contacts in site C.

Specific protein–ligand contacts arise due to multiple weak, low-energy (1–5 kcal/mol), non-covalent interactions, such as H-bonds, ionic forces, and hydrophobic forces at short distance ranges that are sufficient for bonding (2.6–3.4 Å) [39]. Protein–ligand interaction diagrams generated by Schrödinger software throughout the 100 ns MD simulation trajectory and normalized on types of non-covalent bonds were used to estimate HAFP–ligand dynamic interaction modes. Here, we estimated (i) the amino acid residues involved in specific types of interactions during more than 50% of the MD simulation time and (ii) the amounts of protein–ligand contacts at each frame of the MD trajectory. Figure 6 shows the amino acid residues involved in HAFP–ligand interactions, with an indication of how long a specific contact or type of non-covalent force were maintained. Here, Tyr174, Ser216, His222, Ile238, Thr239, His266, Asp478, Ile479, and Cys269–Cys277 disulfide bonds were involved in HAFP–ligand binding in site A. For example, HAFP–E2 interactions were maintained by Tyr174, Leu219, Lys242, Val262, and His266. Here, His266 was involved mainly in H bonding, while Lys242 formed any of four types of non-covalent interactions with the ligand (Figure 6A). His222, Thr239, Asp478, and Ile479 were in contact with DES in this site during more than 50% of the MD simulation time (Figure 6B).

Site B involves Leu138, His170, Phe172, Ser217, Gln221, and Lys453 residues in HAFP–ligand binding. Here, the HAFP–E1 interaction diagram shows that after 15 ns MD simulation, estrone moved from His170, Ser217, and His483 to Lys138, Phe172, and Lys453 (Figure 6C), however the same forces were maintained. HAFP–endoxifen contacts in the same site were maintained by Leu138, Arg169, Ser217, and Lys543 residues (Figure 6D). In site C, HAFP–afimoxifene binding was enabled, mostly by Thr132, Phe172, Gln221, and Lys228 residues, which maintained contact with the ligand for more than half of the MD simulation (Figure 6E). As for HAFP–tamoxifen interaction in this site, in addition to Phe172, Lys129 instead of Lys228 and disulfide bond Cys224–Cys270 were involved (Figure 6F). 

**Figure 7:**
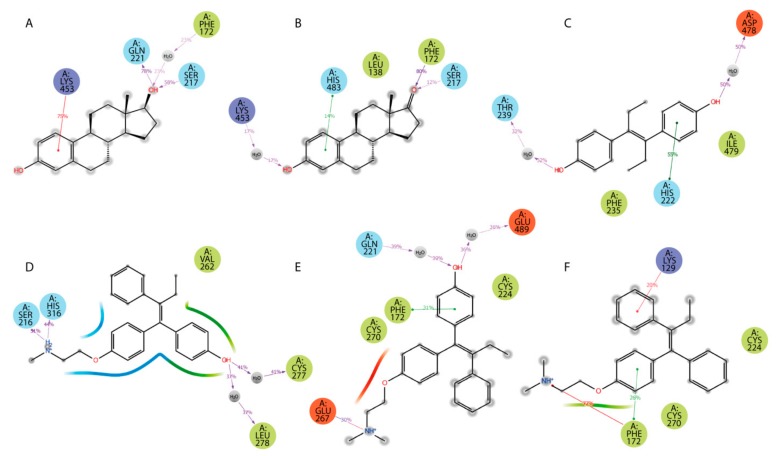
Types of non-covalent forces between functional groups of a ligand and HAFP in the most stable complexes throughout the 100 MD simulation: (**A**) E2 in site B, (**B**) E1 in site B, (**C**) DES in site A, (**D**) endoxifen in site A, (**E**) afimoxifene in site C, (**F**) tamoxifen in site C. Residues are shown as follows: green = hydrophobic; blue = polar; red = negatively charged; violet = positively charged; grey = water (solvent exposure). Interactions are shown as follows: red = π cation; green = π–π stacking; purple = hydrogen bonding.

Additionally, ligand-centered interaction maps (Figure 7) were generated to identify types of non-covalent interactions between functional groups of a ligand and a definite amino acid residue in HAFP. Figure 7A illustrates that HAFP–E2 interactions in site B are mostly caused by H bonds between Gln221 and Ser217 to the OH group at the C17 atom of E2 along with π cation interaction between the aromatic A ring of E2 and the positively charged ε-amino group of Lys453. The water bridge between Lys453 and the OH group at the C3 atom of E1 and the hydrophobic pocket of Leu138 and Phe172 are important in HAFP–E1 interaction in the same site (Figure 7B). In site A, due to its two OH groups, DES interacts with Asp478 and Thr239 residues through water bridges and forms π–π stacking to His222 due to an aromatic ring supported by a hydrophobic pocket consisting of Phe235 and Ile479 (Figure 7C). Endoxifen involves its side chain ^+^NH_2_ group for H bonding with Ser216 and His316. Additionally, it interacts with the Cys269–Cys277 disulfide bond and the hydrophobic pocket of Val262 and Leu278 residues in the binding site (Figure 7D). 

Afimoxifene interacts with HAFP, mostly through its aromatic ring, which is involved in π stacking interactions with Phe172 and the Cys224–Cys270 disulfide bond in site C. Additionally, the ligand OH group is involved in water-mediated H bonding of Gln221 and Glu489, while its side chain NH^+^ group can form salt bridges, H bonds, and water bridges with Glu267 (Figure 7E). Tamoxifen interactions with site C were caused mostly by π cation and π–π stacking bonds to Phe172 or Lys129, as well as hydrophobic interactions with Cys224–Cys270 disulfide (Figure 7F). 

### 2.4. Effects of In Silico Point Amino Acid Substitutions

**Figure 8:**
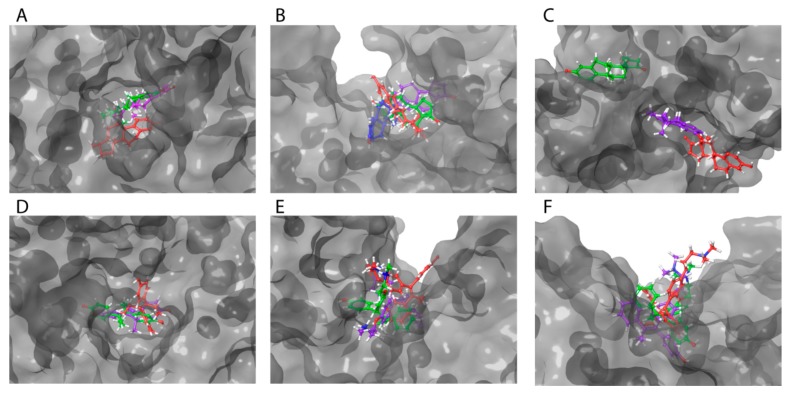
Superimposition of ligands in optimized HAFP–ligand complexes with mutated binding sites. Ligands in all complexes with no mutated HAFP binding sites are shown in green color: 17β-estradiol (**A**) in site A (a tunnel) with substitutions Lys242Leu (violet) and His266Leu (red); (**B**) in site B with substitutions Ser217Ala (violet), Lys453Leu (red), and Gln221Val (blue); estrone in site B (a groove), (**C**) with substitutions Leu138Ser (violet) and Phe172Ala (red); DES in site A (**D**) with substitutions His222Leu (violet) and Asp478Ala (red); endoxifen in site B (**E**) with substitutions: Ser217Ala (violet) and Lys453Glu (red); afimoxifene in site C (**F**) with substitutions Phe172Ala (violet) and Cys224Ala (red).

Table 3 demonstrates impacts of point substitutions of key amino acid residues involved in HAFP–ligand interactions performed in silico in all three putative binding sites identified in our work. Two of the five substitutions performed in HAFP–E2 complexes His266Leu and Lys453Leu remarkably improved MM/GBSA ΔG_bind_ values, while one substitution, Gln221Val, increased the binding energy. Indeed, His266Leu substitution performed in site A caused improvement of the binding pose of 17β-estradiol in this site (Figure 8A). However, diffusion of the ligand from its binding pose in site B was observed, resulting from Gln221Val substitution (Figure 8B), indicating that Gln221 has a key role in HAFP–E2 interaction. 

To confirm the roles of amino acid residues in HAFP–estrone binding, we performed the following substitutions: Leu138Ser, His170Leu, and Phe172Ala in site B. Values of ΔG_bind_ decreased and consequently the affinity of estrone binding to HAFP decreased after all substitutions. Indeed, these substitutions caused estrone diffusion from its binding site (Figure 8C), suggesting the importance of Leu138, His170, and Phe172 in HAFP–estrone binding. Both substitutions performed for HAFP–DES complexes in site A caused a decrease in ligand binding affinities. This was confirmed by visualization of the ligand binding, especially for Asp478Ala replacement, which caused re-arrangement of DES in the binding site (Figure 8D). 

All substitutions in the HAFP–endoxifen complex resulted in increases in ΔG_bind_ values (Table 3), indicating key roles of all replaced residues as well as the Cys269–Cys277 disulfide bond in protein–ligand interactions. However, visualization of the complexes showed that (Figure 8E) only Lys453Glu substitution in site B caused ligand diffusion from its binding site, suggesting a key role of the π cation interaction between the Lys453 side chain and two aromatic rings of endoxifen. All substitutions performed for HAFP–afimoxifene and HAFP–tamoxifen complexes in site C also caused decreases in binding affinities, except Lys228Leu, which improved affinity of afimoxifene binding to HAFP. As a result of Lys228Leu replacement, π cation interaction was replaced by π–π stacking between aromatic rings of Phe172 and afimoxifene, indicating a key role of Phe172. Visualization of the afimoxifene binding in site C showed that both Cys224Ala and Phe172Ala substitutions caused diffusion of the ligand from its binding site, confirming key roles of both Phe172 and Cys224–Cys270 disulfide in the interaction (Figure 8F). 

## 3. Discussion

There are sufficient identity degrees between HAFP and HAS of up to 40% between their full length sequences and approximately 31%, 41%, and 47% between domains I, II, and III, respectively. This makes it possible to construct a 3D model of HAFP based on homology with HSA, for which numerous experimentally resolved structures can be found in the PDB database. Validation of the HAFP 3D model structure created in our study based on homology with HSA showed its high quality and reliability, enabling its usage for molecular docking of ligands, three estrogens, and three antiestrogens in order to identify their binding modes and affinities to the protein. Importantly, the identity degrees between HAFP and HSA domains II and III, where principal ligand binding sites are located, are sufficiently higher than that between domain I.

Earlier, we reported on the construction of the HAFP 3D model structure using Modeller software and based on homology with the same template, HSA (PDB ID 1E78) [35]. In our previous work, we studied binding of a single ligand, DES, to a HAFP modeled structure. This was dictated by experimental data obtained by our group, which demonstrated the high binding efficiency of immobilized DES to HAFP [30]. Molecular docking of DES to the HAFP 3D model and subsequent MD simulation of the obtained complex allowed identification of amino acid residues involved in HAFP–DES interaction. These included Leu138 and residues of the main α helix located in domain III of HAFP and encompassing residues from 446 to 490, which corresponded to binding site B in our present study, located at the bottom of the cavity formed between domains I and III. 

However, the purpose of the present study was the more comprehensive and comparative investigation of HAFP binding affinities of 6 ER agonists and antagonists. SiteMap algorithm predicted 14 potential ligand binding sites in HAFP, and we performed docking all ligands into each predicted site. Based on analysis of ligand-docked scoring functions followed by MD simulation-based ΔG_bind_ energy calculation by MM/GBSA rescoring method, we found that the three most potent binding sites differed by their ligand binding affinities. In all 3 binding sites, designated as A, B, and C, the scoring function values were better for HAFP–antiestrogen complexes than those for HAFP–estrogen complexes. Among antiestrogens, the best scoring functions were observed for docked endoxifen, while 17*β*-estradiol had the best docked scoring functions among estrogens. Ligand-docked scoring functions were better in sites A and C as compared to site B, indicating that the two first sites are high-affinity ones, while the latter is a low-affinity site.

AFP from different mammalian species has been shown to demonstrate similar structural features, activities, and functions. For example, both rodent and human AFPs can bind estrogens, retinoids, bilirubin, and fatty acids [40,41]. Additionally, there are sufficiently high sequence identity percentages (65% and 66%) in HAFP–MAFP and HAFP–RAFP pairs, respectively. Identity degrees between domains I, II, and III of HAFP and rodent AFPs are about 58.5%, 67.0%, and 71.3%, respectively. This makes it possible to compare ligand binding affinities of rodent and human AFPs. Under in vitro conditions, binding of free estrogens to HAFP has not been observed. However, ΔG_exp_ values calculated based on experimentally obtained [42,43,44,45,46] rodent AFP estrogen binding K_a_ values (Tables S1 and S2) show that 17*β*-estradiol has the highest binding affinity and DES has the lowest affinity to both rat and mouse AFPs, which is in agreement with our findings.

We found that HAFP binding affinities for all estrogens were lower than those of antiestrogens, being highest for the HAFP–endoxifen complex in site A and HAFP–afimoxifene complex in site C. Ligand efficiencies were also highest for the same complexes, being lower for estrogens than for antiestrogens. Binding of estrogens to HAFP was mostly provided in our study by van der Waals forces, while both hydrophobic and electrostatic interactions were almost equally involved in HAFP–antiestrogen binding. This shows that the aromatic A ring of estrogens is a key player in HAFP–estrogen interactions, while various factors such as aromatic rings, OH groups, and positive charges on N-atoms in side chains of antiestrogens play key roles in their interactions with proteins. 

Differences in the affinities of estrogens and antiestrogens can be explained by their structural distinctions and amounts of their rotational bonds (Figure 2 and Table 1). Rigid structured estrogens containing four condensed rings showed worse scoring functions for their binding to HAFP as compared to antiestrogens, which are composed of three aromatic rings linked by relatively flexible hydrocarbon linker. The best docked poses among antiestrogens was observed for endoxifen, which can be explained by its distinct structural features, such as the presence of the OH group at the C4 position, which is lacking in tamoxifen, and by the absence of a second *N*-linked CH_3_ group that exists in afimoxifene. 

To confirm the roles of definite amino acid residues in HAFP–ligand interactions revealed by ligand docking and subsequent 100 ns MD simulation, we performed in silico point amino acid substitutions. We found that Thr132, Leu138, His170, Phe172, Ser217, Gln221, His266, His316, Lys453, and Asp478 residues, along with two disulfide bonds (Cys224–Cys270 and Cys269–Cys277), have key roles in both HAFP–estrogen and HAFP–antiestrogen binding.

Generally, point amino acid substitutions do not trigger changes in most dihedral angles in a protein or subsequently the entire protein conformation [47,48]. Accuracy in prediction of the role of residue substitutions in protein functions depends on residue properties such as size, hydrophobicity, and charges (i.e., its type and microenvironment). In our study, we revealed that replacement of amino acid residues involved in protein–ligand interactions allows the same type of ligand movement interaction to be conserved to another residue with the same physicochemical properties. This can be achieved through the ligand movement to another residue with the same physicochemical properties. Despite conformational changes in the AFP molecule having been experimentally shown to have a role in its function [49], further investigations are needed to elucidate roles of ligand binding and amino acid substitutions in changes of binding site geometry and volume. 

## 4. Materials and Methods

### 4.1. HAFP Homology-Based Modeling

#### 4.1.1. Template Identification

The PDB database [33] was searched for experimentally obtained 3D structures of SA family proteins to be used as potential templates for HAFP 3D model building. The HAFP sequence (ID: P02771) was retrieved in FASTA format from Uniprot knowledge base [50] and aligned to primary structures of candidate templates using the BLAST algorithm [51]. The crystal structure of human SA (HSA) obtained at a resolution of 2.6 Å (PDB ID: 1E78) was selected as the best template, with a sequence identity of 39% to HAFP. The edition of the target template alignment was performed using the Prime STA method along with secondary structure element prediction using SSpro utility applied in Prime package, version 5.2. 

#### 4.1.2. Model Generation and Validation 

Protein Preparation Wizard in Schrödinger software version 2018-2 [52] was used to automatically import pdb files of the template and to make all necessary corrections in its structure, such as adding missing hydrogen atoms and determining optimal protonation states. For computational construction of the HAFP homology-based 3D model, we used the highly accurate, fully integrated Prime protein structure prediction suite in Schrödinger software [53]. Visualization of structure-related molecular properties and image presentation were performed through application of the Maestro suite, version 11.6 [54].

The quality and accuracy of the obtained model was validated used several methods, including residue-by-residue geometry and stereochemistry assessment based on Ramachandran map analysis and the PROCHECK algorithm [55]. Further, the generated model was subjected to the refinement procedure, which allowed checking and correction of any inconsistencies in the obtained structure, such as incorrect bond orders, missing hydrogen atoms, and orientation of different functional groups in amino acids. Additionally, to evaluate the stability of the constructed model, calculations of root mean square deviation (RMSD) were performed for the protein backbone and Cα atoms, along with root mean square fluctuations (RMSF) to assess flexible regions during 200 ns MD simulation. 

#### 4.1.3. Model Relaxation and Optimization

The constructed model was subjected to an optimization process during the 200 ns MD simulation through the application of the OPLS3e force field [56] in an explicit water environment generated using the TIP3P water model. For MD simulation, the Desmond program package version 5.4 [57] in Schrödinger software was implemented. MD simulation was carried out in periodic boundary conditions created using orthorhombic boxes, the sizes of which were calculated by buffer method at distances of 10 Å along each dimension. To provide a physiological iso-osmotic environment, 0.15 M NaCl solution was applied. A NPT ensemble was implemented with a Nose–Hoover thermostat [58] to maintain constant temperature equal to 310 K and a Martyna–Tobias–Klein barostat [59] to maintain pressure at 1.01325 bar. Cut-off radii of 9.0 Å were set for non-bonded interactions, and the particle mesh Ewald (PME) method was used to treat long-range electrostatic interactions. The SHAKE algorithm was employed to constrain hydrogen-heavy atom bond lengths. A default relaxation protocol was used before the main simulation. The recording interval was equal to 10 ps, resulting in a total of 20,000 frames. 

### 4.2. Molecular Docking and Scoring 

#### 4.2.1. Ligand Preparation

Structures of ligands (Figure 2) were retrieved from the PubChem database [60]: 17β-estradiol (ID: 5757), estrone (ID: 5870), DES (ID: 448537), tamoxifen (ID: 2733526), endoxifen (ID: 10090750) and afimoxifene (ID: 449459). For accurate 3D ligand model generation and conformational sampling, all ligands were subjected to structure refinement, tautomeric and ionization state prediction, and an optimization procedure using LigPrep suite [61] in Schrödinger software with application of the OPLS3e force field. Protonated states were generated at pH 7.4 ± 1.0 with implementation of the Epik algorithm version 4.4 [62]. For each ligand, 32 possible stereo-isomeric forms were set. 

#### 4.2.2. Identification of Protein–Ligand Binding Sites and Grid Generation

SiteMap suite [63] in Schrödinger software was exploited for accurate identification and evaluation of putative estrogen and antiestrogen binding sites in HAFP. Receptor grid generation was used to construct 36 Å docking grids, which were centered to cover all 14 protein–ligand binding sites predicted by SiteMap suite. The van der Waals radii were scaled at 1.0 and with a partial charge cut-off of 0.25, while other atoms were free of scaling.

#### 4.2.3. Docking Protocol

Ligand-docked poses were generated by searching the most populated conformations for each protein–ligand complex. Schrödinger Glide suite version 7.9 [64] was employed with an extra precision ligand docking protocol to commence HAFP–ligand docking, followed by calculation of scoring functions. Here, ligands were treated as flexible, while the conformer generation procedure included (non-ring) nitrogen inversions and ring conformations. No rotation was allowed for the rotatable protein bonds.

#### 4.2.4. Scoring Functions

To evaluate ligand-docked poses, gscore and emodel scoring functions were calculated in the extra precision Glide score (XP GScore) tool of Schrödinger software. The protein–ligand complexes with the best scoring functions were selected for further MD simulation. 

### 4.3. MD Simulation of Protein–Ligand Complexes

The final ligand-docked poses with the best scoring functions were subjected to 100 ns MD simulation in an explicit water environment generated using the TIP3P water model. The Schrödinger Desmond suite version 5.4 [57] was used with a writing interval of 20 ps for each frame, along with the OPLS3e force field [56]. Periodic boundary conditions with box sizes of 20Å at each dimension were calculated by buffer method. The other parameters of the MD simulation protocol were the same as described earlier for the HAFP model optimization. The quality of the MD simulation was assessed by Simulation Interactions Diagram Reports generated by Schrödinger software for mapping and analysis of geometry of ligand binding sites and elucidation of amino acid residues involved in HAFP–ligand interactions. The reports include: (i) protein information, including secondary structure elements; (ii) ligand information, including torsion angle profile and surface properties; (iii) RMSD and RMSF plots; and (iv) comprehensive analysis of protein–ligand contacts.

### 4.4. RMSD and RMSF Calculations for Protein–Ligand Complexes

RMSD calculations for protein backbones and Cα atoms during 100 MD simulation of HAFP–ligand complexes were used to get an insight into conformational changes during protein–ligand interactions, both with and without amino acid substitutions. RMSD analysis of a ligand when in a protein–ligand complex was performed to judge the stability of the ligand in its binding site and its ability to diffuse from the binding site during MD simulation or as a result of amino acid replacements.

To characterize local changes in the protein backbone during 100 ns MD simulation and residue-by-residue fluctuations in the HAFP molecule during its interaction with a ligand, RMSF calculations were made [65]. Protein RMSF plots were superimposed on secondary structure elements to evaluate their stability.

### 4.5. In Silico Point Amino Acid Substitutions

To confirm the roles of amino acid residues involved in HAFP–ligand interactions revealed by ligand docking and subsequent 100 ns MD simulation, we computationally performed point substitutions of amino acid residues in putative ligand binding sites. Mutate Residues tool of the Protein Preparation and Refinement suite in Schrödinger software was employed to perform residue substitutions in each of the three ligand binding sites found in HAFP. Further, the mutated structures were subjected to 100 ns MD simulation using the same protocol as described above. Assessment of the point residue substitution impact on HAFP–ligand interactions was carried out in several ways, including through: (i) generation of Simulation Interactions Diagram Reports after each substitution; (ii) superimposition of binding poses of the same ligand in the same site with mutated residue; and (iii) calculation of changes in binding free energy (ΔG_bind_).

### 4.6. Binding Free Energy Calculations by MM/GBSA Rescoring Method

Calculation of binding free energy (ΔG_bind_) values was exploited to estimate in silico ligand binding affinities. For this purpose, the MM/GBSA rescoring method was employed with the use of the Prime module [53] in Schrödinger software to estimate energy properties and contributions from different energy terms [66,67,68].

MM/GBSA rescoring was performed for initial ligand-docked poses with best scoring functions and for each protein–ligand complex during 100 ns MD simulation, with and without amino acid substitutions. In total, 1000 snapshots were generated from each MD simulation trajectory and average ΔG_bind_ values were calculated [69]. The free energy changes during protein–ligand interactions were calculated with the use of the VSGB solvent model. Before calculations, all counter ions and water molecules were deleted from the system. 

Binding free energy values were calculated according to the following equation (Equation (1)):

(1) MMGBSAΔGbind=Gcomplexoptimized−Gproteinoptimized+ Gligandoptimized 

The free energy of each state (i.e., of complex, protein, and ligand states) was estimated by accounting for molecular mechanics energies, solvation energies, and entropic terms, as follows (Equation (2)):

(2) $$G=G_{int}+G_{Coulomb}+G_{vdW}+G_{GB}+G_{lipo}-TS$$

where G_int_, G_Coulomb_, and G_vdW_ are standard MM energy terms for bonds (covalent, angle, and dihedral), Coulomb (electrostatic) interactions, and van der Waals interactions, respectively. G_GB_ and G_lipo_ are polar and non-polar (lipophilic) contributions to solvation free energies, while T is the absolute temperature and S is an entropy value. The polar contribution (G_GB_) was calculated using the generalized Born model, while non-polar contribution (G_lipo_) was estimated based on the solvent accessible surface area (SASA).

The ligand efficiency (LE) values were calculated as follows (3):

(3) LE=lnΔGbindoptimizedn

where n is the amount of heavy ligand atoms.

To calculate experimental binding free energies (ΔG_exp_), dissociation constant (K_d_) or IC_50_ values obtained from literature data were used. Here, ΔG_exp_ was assessed as follows (Equation (4)):

(4) $$\Delta G_{exp}=RTlnK_{d}\;\;\;\mathrm{OR}\;\;\;G_{exp}=RT\;lnIC_{50}$$

## 5. Conclusions

In our study, we showed that three major binding sites differed by the values of their binding free energies, which can be distinguished in HAFP. Homology-based modeling, molecular docking, and MD simulation studies allowed the topology of the protein surface and geometries of ligand binding sites to be elucidated, along with the amino acid residues involved in HAFP–ligand interactions. Point substitutions of the residues performed in silico allowed those residues that can have key roles in HAFP–ligand binding to be distinguished. Our results are in agreement with previously obtained experimental data. However, further investigations using novel experimental and computational approaches are needed. 

## Figures and Tables

**Table 1:** Scoring functions for the best docked HAFP–estrogen and HAFP–antiestrogen complexes. DES = diethylstilbestrol.

Docked Ligand	Amount of Rotational Bonds	Binding Site	Glide Gscore	Glide Emodel
17β-estradiol	2	A	−5.420	−49.732
		B	−4.807	−46.737
		C	−5.384	−35.722
Estrone	1	A	−4.109	−45.332
		B	−4.054	−45.389
		C	−4.408	−38.067
DES	6	A	−4.934	−34.058
		B	−3.126	−33.573
		C	−3.440	−37.538
Afimoxifene	9	A	−5.932	−57.375
		B	−3.840	−55.881
		C	−5.308	−54.272
Endoxifen	9	A	−6.322	−61.786
		B	−4.247	−50.983
		C	−5.768	−59.989
Tamoxifen	8	A	−4.805	−53.899
		B	−2.976	−43.853
		C	−4.169	−51.536

**Table 2:** Values of calculated binding energies and energy terms for HAFP–ligand docked poses and optimized by100 ns MD simulation HAFP–ligand complexes. MM/GBSA = molecular mechanics/Generalized Born surface area; ΔG = Free energy change.

Complex	MM/GBSA ΔG_bind_ Docked Poses	MM/GBSA ΔG_bind_ Optimized Complexes	ΔG_Coulomb_ Optimized	ΔG_vdW_ Optimized	ΔG_GB_ Optimized	ΔG_lipo_ Optimized	Ligand Efficiency
**Site A**							
**HAFP–17β-estradiol**	−35.03	−38.29	−9.55	−33.20	20.50	−15.99	−9.58
**HAFP–DES**	−49.14	−50.99	−10.68	−39.69	20.06	−21.11	−12.76
**HAFP–endoxifen**	−64.50	−64.18	−42.01	−47.54	56.66	−28.06	−14.81
**HAFP–afimoxifene**	−69.34	−57.23	−36.93	−47.05	49.76	−25.18	−13.10
**HAFP–tamoxifen**	−30.53	−48.09	−32.86	−42.11	45.60	−19.74	−11.10
**Site B**							
**HAFP–17β-estradiol**	−42.85	−44.22	−15.23	−29.95	15.18	−13.00	−11.05
**HAFP–estrone**	−42.51	−50.31	−10.81	−36.08	13.86	−16.32	−12.59
**HAFP–endoxifen**	−55.15	−59.61	−41.92	−43.93	43.95	−18.22	−13.76
**Site C**							
**HAFP–endoxifen**	−59.13	−45.38	−39.41	−39.13	45.95	−15.91	−10.47
**HAFP–afimoxifene**	−53.80	−61.32	−39.94	−47.06	46.92	−22.23	−14.04
**HAFP–tamoxifen**	−52.68	−58.74	−33.62	−45.91	41.10	−23.04	−13.56
**HAFP–DES**	−44.47	−36.33	−9.55	−22.88	14.52	−17.71	−9.09

**Table 3:** MM/GBSA free energy changes and energy terms resulting from point residue substitutions in optimized HAFP–ligand complexes.

Complex	Binding Site	Substitution	MM/GBSA ΔG_bind_^a^	ΔG_Coulomb_	ΔG_vdW_	ΔG_GB_	ΔG_lipo_	Ligand Efficiency
**HAFP–** **17β-estradiol**	A	Lys242Leu	−39.3628 (−38.2924)	−7.2468	−34.8330	22.0165	−19.3103	−9.8512
		His266 Leu	−42.5281 (−38.2924)	−7.8309	−35.1273	19.5618	−19.4308	−10.6434
	B	Ser217Ala	−45.6250 (−44.2185)	−16.5316	−30.8540	16.4249	−13.7538	−11.3184
		Gln221Val	−41.9187 (−44.2185)	−11.0667	−31.4626	16.5076	−14.9278	−10.4909
		Lys453Leu	−51.1980 (−44.2185)	−20.9175	−30.2452	17.0518	−16.4198	−12.8132
**HAFP–** **estrone**	B	Leu138 Ser	−47.0881 (−50.31)	−11.0476	−37.6467	16.8381	−15.1892	−11.7846
		Phe172Ala	−32.3050 (−50.31)	−7.8493	−30.9881	14.3777	−8.2677	−8.0849
		His170 Leu	−40.3051 (−43.88)	−8.1035	−33.2855	15.8254	−14.8531	−10.0870
**HAFP–DES**	A	Asp478Ala	−36.5685 (−51.00)	−11.7190	−30.5551	20.7434	−15.9298	−9.1519
		His222Leu	−48.0213 (−51.00)	−11.2453	−34.2183	16.6168	−20.4277	−12.0181
**HAFP–** **endoxifen**	A	Cys277Val	−54.0787 (−64.18)	−36.8418	−45.6549	55.1793	−25.6210	−12.4830
		His316Leu	−54.1489 (−64.18)	−38.4019	−45.9868	54.6261	−23.2189	−12.4991
	B	Lys453 Glu	−50.4113 (-59.61)	−36.0847	−40.3290	45.4848	−19.6389	−11.6364
		Ser217Ala	−50.5624 (−59.61)	−43.0608	−41.9079	51.3839	−17.3874	−11.6713
		Lys228Glu	−57.2781 (−59.61)	−42.2583	−41.4879	43.1563	−17.1490	−13.2215
**HAFP**– **afimoxifene**	C	Lys228Leu	−66.8559 (−50.07)	−42.6339	−48.0607	51.2632	−27.7603	−15.3083
		Thr132 Val	−44.5842 (−50.07)	−34.4384	−41.7174	45.6890	−16.7658	−10.2087
		Cys224Ala	−49.2394 (−61.32)	−40.4615	−39.3824	44.3091	−16.9701	−11.2746
		Phe172Ala	−51.4201 (−61.32)	−41.2409	−45.8675	49.0546	−16.9381	−11.7739
**HAFP**– **tamoxifen**	C	Phe172Leu	−51.2034 (−58.74)	−32.8152	−45.6450	43.1294	−17.2336	−11.8192
		Cys224Val	−41.0369 (−58.74)	−27.3946	−36.2815	36.6834	−16.0279	−9.4725

^a^ Values of MM/GBSA/ΔG_bind_ before substitutions are given in brackets.

## Supplementary Materials

Supplementary materials can be found at https://www.mdpi.com/1422-0067/21/3/893/s1.

## Abbreviations

HAFP	human α-fetoprotein
HSA	human serum albumin
E2	17β-estradiol
DES	diethylstilbestrol
