# Supplementary material for: Elucidating Binding Sites and Affinities of ERα Agonists and Antagonists to Human Alpha-Fetoprotein by In Silico Modeling and Point Mutagenesis
Source: Int J Mol Sci. 2020 Jan 30;21(3):893. doi: 10.3390/ijms21030893 (PMC7036865; doi:10.3390/ijms21030893)
Supplement: Supplementary file 1 [file ijms-21-00893-s001.pdf]

Supplementary materials

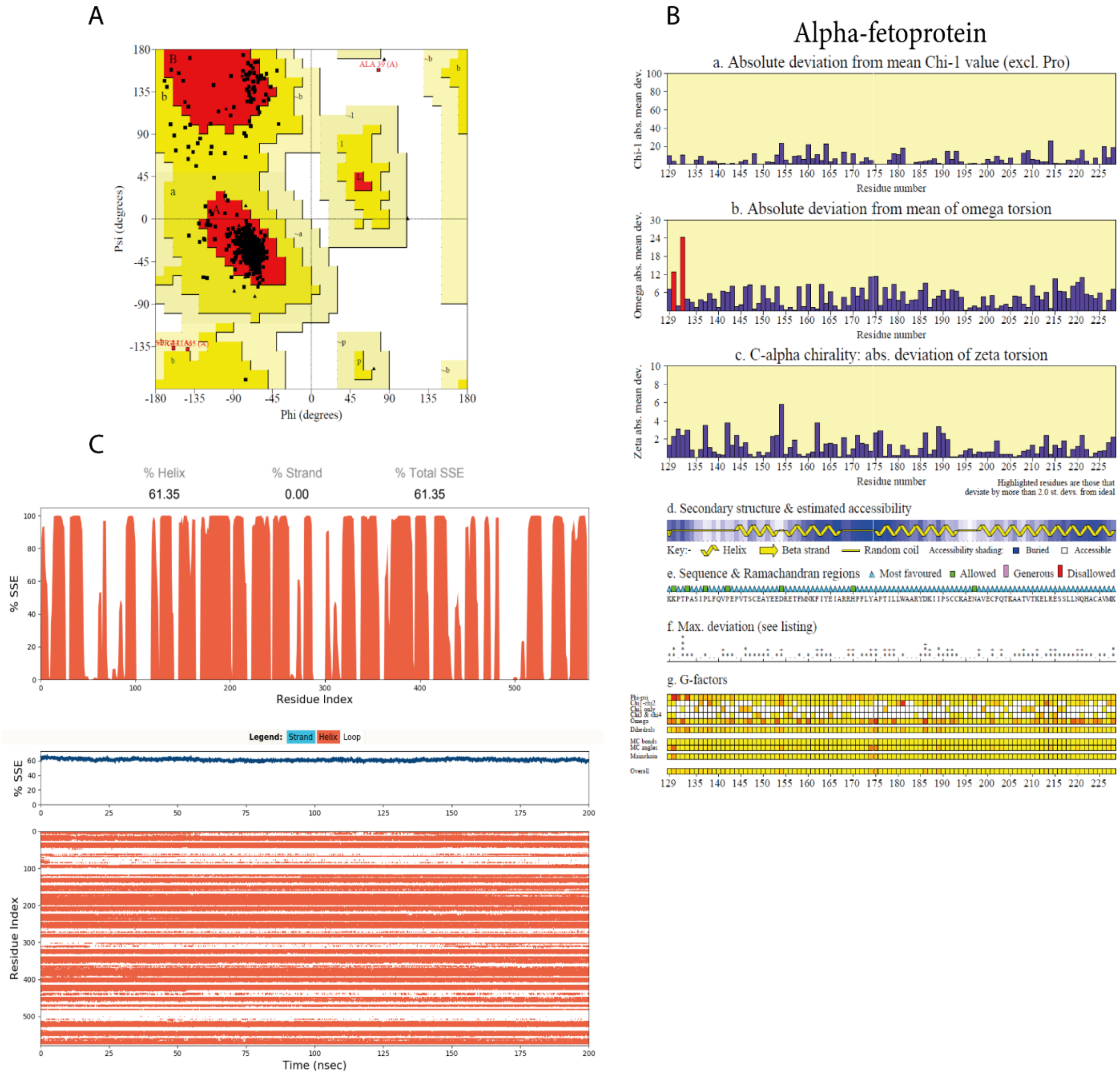

Figure S1. Validation of HAFP 3D model structure obtained based on homology with HSA. (A) Ramachandran map for the main chain conformation shows that 99.5% residues (black dots) are located in favored (red) and allowed (yellow) regions. (B) Graphs and diagrams of stereo-chemical properties of individual residues demonstrate high quality of the model structure. (C) Secondary structure element (SSE) diagrams demonstrate stability of α-helical regions.

Table S1. Experimental binding affinities of estrogens to rat AFP

Ligand	K_a RAFP (M^{-1})	K_d RAFP (M)	ΔG_{RAFP} (kcal/mol)	References
**17β-estradiol**	From 9.3×10^8<br>To 11.4×10^8	From 0.107×10^{-8}<br>To 0.088×10^{-8}	From -12.232<br>To -12.347	[31]
**17β-estradiol**	From 0.6×10^8<br>To 1.4×10^8	From 1.667×10^{-8}<br>To 0.714×10^{-8}	From -10.606<br>To -11.106	[42]
**17β-estradiol**	$2.83 \pm 0.78 \times 10^8$	0.353×10^{-8}	-11.525	[43]
**17β-estradiol**	5.0×10^7	0.200×10^{-7}	-9.135	[44]
**Estrone**	$5.51 \pm 1.01 \times 10^8$	0.182×10^{-8}	-11.917	[43]
**Estrone**	9.0×10^7	0.111×10^{-7}	-10.847	[44]
**DES**	1.5×10^6	0.667×10^{-6}	-8.452	[45]

Table S2. Experimental binding affinities of estrogens to mouse AFP

Ligand	K_a MAFP (M^{-1})	K_d MAFP (M)	ΔG_{MAFP} (kcal/mol)	References
**17β-estradiol**	0.8×10^8	1.25×10^{-8}	-10.777	[46]
**DES**	0.2×10^7	5.00×10^{-7}	-8.592	[46]
